# A case report of pediatric-onset MS associated uveitis

**DOI:** 10.1186/s12348-024-00405-1

**Published:** 2024-05-29

**Authors:** Zahra Mahdizad, Mohammad Zarei, Hanieh Fakhredin, Reza Samiee, Hora Heidari, Nazanin Ebrahimiadib

**Affiliations:** 1 Ophthalmology Department, Retina Service, Farabi Eye Hospital, Tehran University of Medical Sciences, Tehran, Iran; 2 Student`S Scientific Research Center, Tehran University of Medical Sciences, Tehran, Iran; 3 Multiple Sclerosis Research Center, Tehran University of Medical Sciences, Tehran, Iran; 4 Ophthalmology Department, Ocular Immunology and Uveitis Foundation, Waltham, MA USA; 5 Retina & Vitreous Service, Farabi Comprehensive Center of Excellence in Ophthalmology, Qazvin Square, Tehran, 1336616351 Iran

**Keywords:** Pediatric-onset MS (POMS), Uveitis, Intermediate Uveitis (IU), Pediatric IU

## Abstract

**Background:**

To report a case of Pediatric-onset MS associated uveitis managed with local and systemic medications.

**Case presentation:**

An 11-year-old boy who was diagnosed with Pediatric-onset MS (POMS) with the first presentation of left optic neuritis in another center, was referred to our clinic with the complaint of non-improved vision in the left eye despite receiving IV 5gr methylprednisolone. After the ophthalmologic examinations, the patient was diagnosed as bilateral POMS-associated intermediate uveitis, and local treatment with corticosteroid was administered to both eyes. He was continued on systemic therapy such as Rituximab and five sessions of plasmapheresis. After four months, the patient's vision improved from FC at 50cm to 9/10 in the left eye. The intensity of intraocular inflammation decreased in both eyes. In fluorescein angiography findings, the optic disc, as well as vascular leakage, subsided bilaterally.

**Conclusion:**

Despite its rarity, POMS-associated uveitis presents a considerable challenge that necessitates the collaborative efforts of neurologists and ophthalmologists to achieve the most effective treatment outcomes.

**Supplementary Information:**

The online version contains supplementary material available at 10.1186/s12348-024-00405-1.

## Background

Intermediate uveitis (IU) is a subtype of uveitis in which the primary sites of inflammation is anterior vitreous and pars plana [1]. Although relatively rare in adult-onset uveitis, IU can affect up to 33% of uveitis cases under the age of 16 [2]. The etiologies of IU may be broadly categorized into infectious or non-infectious [2]. Pediatric IU differs from adult IU in that there is frequently an absence of an underlying systemic condition [2, 3].

One of the most well-known non-infectious etiologies in adult IU is multiple sclerosis (MS). The reported prevalence of uveitis in MS ranges from 0.65% to 1.1%, and in those with uveitis, MS can be discovered in 0.9% to 1.7% of cases [4]. Due to overall rarity of MS in individuals under the age of 18 [5], the exact prevalence of uveitis in this age group has not been investigated thoroughly. In a particular study, the occurrence rate of uveitis within the POMS group has been approximated to be around 9% [6] which seems to be higher than the prevalence of uveitis in adult MS.

Herein, we report a child with the diagnosis of MS and associated optic neuritis and uveitis.

## Case presentation

An 11-year-old boy who was diagnosed with MS was referred to our clinic with the complaint of reduced vision in his left eye. He had noticed a decreased vision in his left eye following a mild head trauma 2 weeks prior to our visit. He was found to have optic nerve swelling and was referred to a neurologist. Neurological examinations were normal. Brain magnetic resonance imaging (MRI) revealed high T2/FLAIR intensity lesions at juxta-cortical, subcortical, and periventricular white matter, as well as corpus callosum, calloso-septal interface and cervical and thoracic spinal cord without restricted-diffusion or enhancement (Supplementary Fig. 1). The diagnosis of pediatric-onset MS (POMS) was established by an MS specialist. Intravenous (IV) methylprednisolone (1g daily for 5 days) was administered, but his vision did not improve prompting referral to our center.

In ophthalmologic examination in our clinic, the patient's visual acuity (VA) was 6/10 in the right and finger counting (FC) at 50 cm in the left eye. Anterior segment examination revealed bilateral mild injection of conjunctiva. There were no cells in the anterior chamber (AC). The intraocular pressure (IOP) measurements were within normal limits. A prominent relative afferent pupillary defect (RAPD) was present in the left eye. Vitreous organization, 2 + vitreous cells and haze, snowballs and snowbanks were observed in both eyes.

In fundus examination, the left optic disc had blurred margins along with peripapillary hemorrhage. The right eye optic nerve examination was unremarkable (Figur 1-a, b). Perivascular sheathing in the peripheral retina could be appreciated in both eyes.

**Fig. 1:**
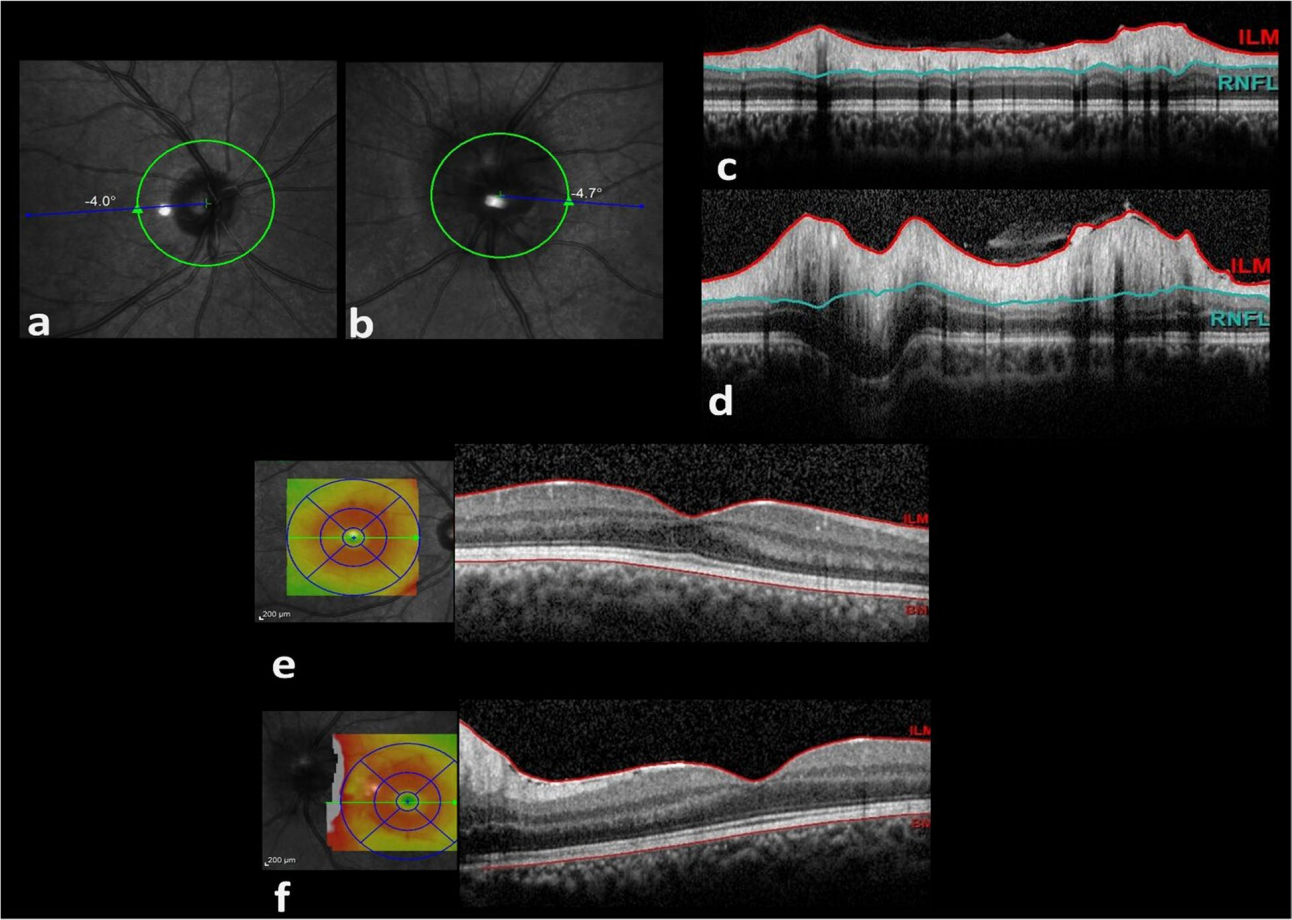
**a**. IR photo of right optic nerve head is unremarkable. **b**. IR photo of left optic nerve head showed optic disc swelling. Corresponding OCT slabs revealed normal RNFL thickness in the right eye (**c**, average general thickness = 149) and generalized increased RNFL thickness in left eye (**d**, average general thickness = 295). Corresponding foveal horizontal OCT b-scans and thickness maps show vitreous cells in OU and increased peripapillary RNFL thickness in OS as well as increased parafoveal retinal thickness in both eyes (**e**, **f**)

**Fig. 2:**
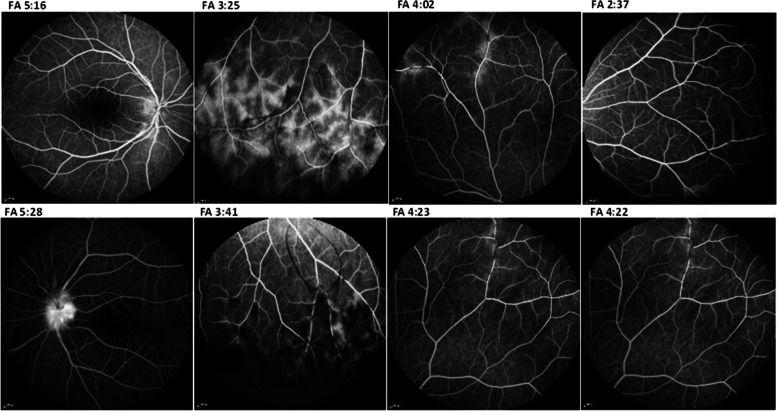
late phase FA revealed mild optic disc and moderate peripheral vascular leakage in the right eye (first raw) as well as severe optic disc and mild peripheral vascular leakage in the left eye (second raw)

Retinal nerve fiber layer (RNFL) optical coherence tomography (OCT) scans revealed remarkable increase in the left eye optic nerve head RNFL thickness (Fig. 1-c, d). Macular spectral domain (SD)-OCT illustrated the posterior vitreous cells; Both eyes exhibited perifoveal non-cystic retinal thickening, which could potentially serve as an indicator of significant peripheral retinal vascular leakage, as suggested by a recent study [7] (Fig. 1-e, f). In fluorescein angiography, optic disc leakage, periphlebitis and vascular leakage in equator and more predominantly in the periphery of the retina were observed bilaterally; no macular leakage was evident.(Fig. 2).

All laboratory testing including PPD, ACE, VDRL and RPR were reported within normal range or non-reactive.

The patient was diagnosed with bilateral POMS associated intermediate uveitis. Considering active uveitis despite receiving high dose systemic corticosteroid, posterior sub-Tenon triamcinolone acetonide (20 mg/0.5 ml) was administered to each eye.

At the same time, the patient was treated with another IV methylprednisolone (1 g daily for 3 days) and also plasmapheresis for five sessions in neurology ward; the treatment was continued with Rituximab (prescribed as 500 mg, IV infusion separated by two weeks and then every 6 months).

**Fig. 3:**
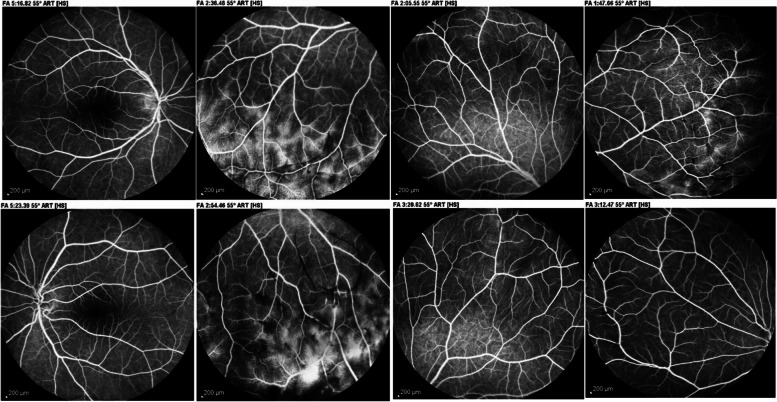
late phase FA in 4th month of follow up showed decrease in the severity of optic disc and peripheral vascular leakage in both eyes

After four months, the patient's vision improved to 10/10 and 9/10 in the right and left eye, respectively. The intensity of vitreous cells and haze decreased to 0.5 + in both eyes. At 4 months follow-up, fluorescein angiography demonstrated that optic disc and vascular leakage had subsided bilaterally (Fig. 3).

## Discussion

Pediatric MS, also referred to as pediatric-onset MS, early-onset MS or juvenile MS, challenges the management of pediatric IU. Less than 10% of patients with MS are under the age of 16, and 1% are even under the age of 10 [8].

The clinical characteristics of MS in children differ from that in adults; as the patients generally experience a more aggressive disease onset and course [9]. The diagnosis can be challenging, and many different diagnostic criteria have been proposed. The criteria developed by the Pediatric International Study Group have been employed by most researchers as well as our neurology colleagues in our institution [10].

The same problem exists for the diagnostic criterion for POMS-associated IU. However, it seems that the standardization of uveitis nomenclature (SUN) working group criteria for adult MS-associated IU can be used with considerations [11].

It should be pointed out that there are currently no distinct clinical or imaging findings that can accurately identify patients with IU who are at a higher risk of developing MS later on. As a result, routine brain MRI is not recommended for patients with IU unless they already display neurological symptoms or signs, or have additional neuro-ophthalmologic signs such as optic neuritis [12].

Acute pediatric demyelination is typically treated with first-line pulse steroid therapy (20 to 30 mg/kg/day (up to 1 g/day) for 3 to 5 days). Second-line therapies such as intravenous immunoglobulin (IVIG- 2 g/kg given over 2–5 days) or plasma exchange (to remove the pathological antibodies, cytokines, and other circulating inflammatory components from the bloodstream) are considered in patients with severe events or with incomplete or no improvement after receiving high-dose pulse steroid treatment [13, 14]. Considering that frequent relapses are correlated with poor prognosis, early institution of disease-modifying therapies for POMS is highly recommended [9]. According to certain specialists, it is advocated that potent immunosuppressant medications be administered initially to halt the progression of the disease and restore the functioning of the immune system. For patients with active disease and a higher likelihood of early disability, more intensive immunosuppressant treatment should be contemplated for a limited duration to attain disease control. Subsequently, a maintenance therapy involving lower-risk therapies should be implemented [15].

The presence of IU in patients with MS does not necessarily warrant the initiation of disease-modifying drugs (DMDs). Instead, the management approach should be customized to address the specific requirements for uveitis treatment. Nevertheless, for patients with MS who are already receiving DMDs, it is advisable to opt for local therapies to address ocular inflammation associated with IU. This approach helps minimize the risk of additional systemic side effects that may arise from immunomodulatory therapy. Of note, treatment with IFNβ, glatiramer acetate, mycophenolate mofetil, natalizumab, alemtuzumab, and anti-CD20 agents is effective in the management of both MS and IU [12].

Managing IU in pediatric patients with MS poses distinct challenges that hinder the establishment of a standardized guideline. The potential systemic side effects of medications on cognitive and physical development cannot be overlooked. Conversely, the utilization of local steroid injections, which are frequently administered in adults to mitigate systemic side effects, may pose the potential risk of glaucoma and cataract in pediatric patients. Moreover, the examination and monitoring of these young patients can be more intricate. Therefore, ophthalmologists must carefully evaluate each patient's unique circumstances and tailor their treatment approaches accordingly.

We successfully managed our patient with active POMS-associated IU who had received high dose systemic steroid therapy, by adjuvant periocular steroid injection. We believe local steroid therapies could be administered to minimize the systemic side effects associated with systemic therapies in pediatric patients who are cooperative during examinations to detect potential ocular side effects such as glaucoma. Nevertheless, it is advisable to adopt a multidisciplinary approach involving both a neurologist and an ophthalmologist to ensure optimal treatment outcomes.

## Conclusion

The diagnosis and treatment of POMS-associated uveitis can present considerable challenges. Given its rarity, timely diagnosis of this condition requires a high index of suspicion. Patients with IU should undergo a thorough evaluation of neurologic and neuro-ophthalmologic signs and symptoms and neuroimaging is highly recommended in patients with positive findings; Additionally, individuals with MS who experience visual symptoms must be promptly examined by an ophthalmologist. To effectively manage patients with POMS and IU, a collaborative approach involving a multidisciplinary team comprising ophthalmologists and neurologists is essential.

### Supplementary Information

Supplementary Material 1. **Supplementary Figure 1.** high T2/FLAIR signal intensity lesions are seen at juxtracortical, subcortical, and periventricular white matter, corpus callosum, calloso-septal interface without restricted-diffusion or enhancement. left optic nerve has abnormal signal intensity and enhancement in favor of optic neuritis. Multiple abnormal signal intensities are seen at cervical and thoracic spinal cord without enhancement.

## Data Availability

The data used in that case report is available from the corresponding author on reasonable request.
