# Supplementary material for: A case report of pediatric-onset MS associated uveitis
Source: J Ophthalmic Inflamm Infect. 2024 May 29;14:21. doi: 10.1186/s12348-024-00405-1 (PMC11136925; doi:10.1186/s12348-024-00405-1)
Supplement: Supplementary file 1 — Supplementary Material 1. Supplementary Figure 1. high T2/FLAIR signal intensity lesions are seen at juxtracortical, subcortical, and periventricular white matter, corpus callosum, calloso-septal interface without restricted-diffusion or enhancement. left optic nerve has abnormal signal intensity and enhancement in favor of optic neuritis. Multiple abnormal signal intensities are seen at cervical and thoracic spinal cord without enhancement. [file 12348_2024_405_MOESM1_ESM.docx]

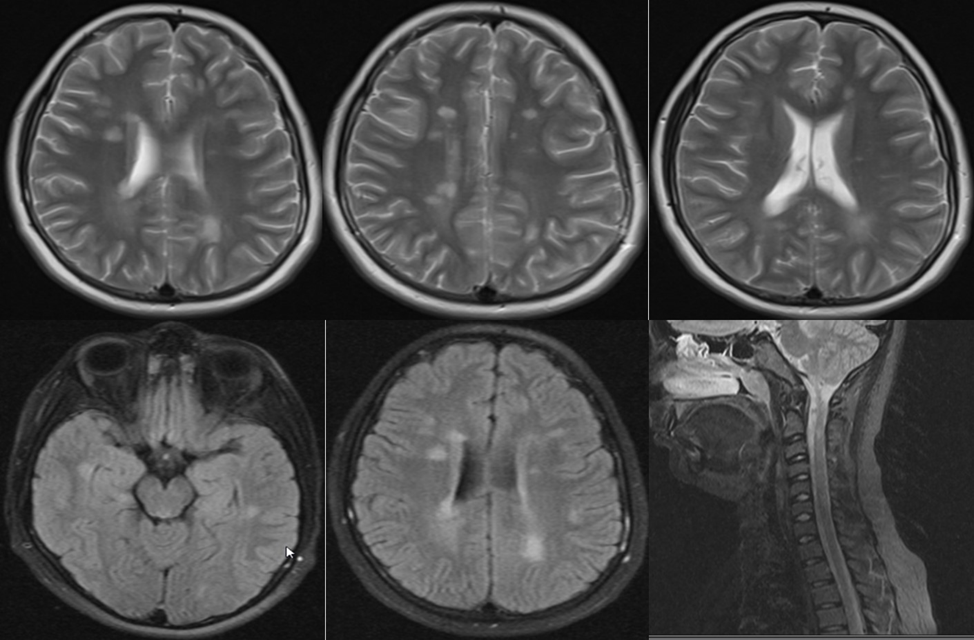


**Supplementary Figure 1- high T2/FLAIR signal intensity lesions are seen at juxtracortical, subcortical, and periventricular white matter, corpus callosum, calloso-septal interface without restricted-diffusion or enhancement. left optic nerve has abnormal signal intensity and enhancement in favor of optic neuritis. Multiple abnormal signal intensities are seen at cervical and thoracic spinal cord without enhancement.**
